# Recent Developments and Applications of Nanosystems in the Preservation of Meat and Meat Products

**DOI:** 10.3390/foods11142150

**Published:** 2022-07-20

**Authors:** Araceli Ulloa-Saavedra, Claudia García-Betanzos, María Zambrano-Zaragoza, David Quintanar-Guerrero, Susana Mendoza-Elvira, Benjamín Velasco-Bejarano

**Affiliations:** 1 Laboratorio de Procesos de Transformación y Tecnologías Emergentes de Alimentos, FES-Cuautitlán, Universidad Nacional Autónoma de México, Cuautitlán Izcalli CP 54714, Edo Mex, Mexico; araceli_ulloa@comunidad.unam.mx (A.U.-S.); idalid_gb@unam.com (C.G.-B.); 2 Laboratorio de Posgrado en Tecnología Farmacéutica, FES-Cuautitlán, Universidad Nacional Autónoma de México, Cuautitlán Izcalli CP 54745, Edo Mex, Mexico; quintana@unam.mx; 3 Laboratorio de Virología, FES-Cuautitlán, Universidad Nacional Autónoma de México, Cuautitlán Izcalli CP 54745, Edo Mex, Mexico; seme@unam.mx; 4 Laboratorio de Química Verde Medicinal, FES-Cuautitlán, Universidad Nacional Autónoma de México, Cuautitlán Izcalli CP 54745, Edo Mex, Mexico; qfbbenjamin.velascob@cuautitlan.unam.mx

**Keywords:** lipid oxidation, shelf life, antimicrobial effect, natural additives, nanosystem

## Abstract

Due to their high water, lipid, and protein content, meat and meat products are highly perishable. The principal spoilage mechanisms involved are protein and lipid oxidation and deterioration caused by microbial growth. Therefore, efforts are ongoing to ensure food safety and increase shelf life. The development of low-cost, innovative, eco-friendly approaches, such as nanotechnology, using non-toxic, inexpensive, FDA-approved ingredients is reducing the incorporation of chemical additives while enhancing effectiveness and functionality. This review focuses on advances in the incorporation of natural additives that increase the shelf life of meat and meat products through the application of nanosystems. The main solvent-free preparation methods are reviewed, including those that involve mixing organic–inorganic or organic–organic compounds with such natural substances as essential oils and plant extracts. The performance of these additives is analyzed in terms of their antioxidant effect when applied directly to meat as edible coatings or marinades, and during manufacturing processes. The review concludes that nanotechnology represents an excellent option for the efficient design of new meat products with enhanced characteristics.

## 1. Introduction

Meats and meat products are foods with high protein content and consumer preference, but they are especially susceptible to microbial growth and lipid oxidation, the leading causes of the deterioration that leads to the rapid loss of important physical, chemical, and sensory properties [1,2]. Hence, the meat industry is continuously searching for new preservation methods that will increase the shelf life of fresh products and aid in developing unique, enhanced derivatives. Emerging approaches such as UV-C irradiation, ultrasound, pulsed electric field, and nanotechnology are some of the methods applied. Nanotechnology is employed mainly to improve the sensory characteristics of fresh meat, and minimally processed, ready-to-eat, meat products [3]. Nanosystems are characterized by sizes <1000 nm in one or more dimensions. For use in food, sizes of 100–500 nm are preferred to reduce the risk of nanosized substances crossing into the bloodstream [4,5]. The properties of nanosystems differ from those of larger-sized systems with similar chemical composition due to their greater surface area per mass. In addition, nanosystems prepared with natural materials are more stable and compatible with biological tissues, so they reduce risks of toxicity [6,7].

Nanotechnology permits incorporating hydrophobic and hydrophilic bioactives and other natural compounds that provide positive effects related to particle size, such as reducing the amount of bioactive ingredients to minimize changes in the sensory characteristics of products, maintain the attractive visual features of food, increase shelf life, and conserve nutritional value, functionality, and stability [8,9], and inhibit the growth of foodborne pathogens [10]. This method also helps incorporate natural additives with antioxidant and antimicrobial activity, modifiers, and texture, flavor, and color enhancers into meat preservation and processing [11,12]. The aim of this review is to discuss recent advances in the preparation, methodology, and application of nano-size ingredients of natural origin in preserving meat and meat products.

## 2. Evolution of Nanotechnology in Food Applications

**Figure 1:**
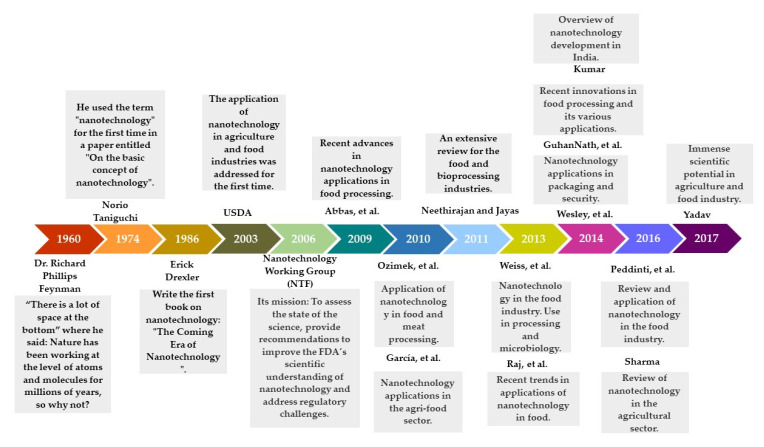
Chronology of nanotechnology applied to food.

The potential use of nanotechnology in food industries has grown in association with increased interest in reducing the use of synthetic additives and other ingredients. But this is not a recent development. In fact, its history began in 1965 when Richard Feynman stated in his speech upon receiving the Novel Prize that “there was too much space at the bottom,” referring to all the possibilities that remained to be investigated. Norio Taniguchi coined the term “nanotechnology” just a few years later, in 1974. Figure 1 shows the evolution of nanotechnology with emphasis on food applications.

## 3. Effects of Nanosystems on Sensory Properties of Food

Nanotechnology in food processing can modify physicochemical properties such as color, flavor, or nutrients to suit industrial requirements [13] and they could have the capacity to mask the adverse effects of unpleasant odors and undesirable flavors, but it is important to be compatible with the quality aspects [14]. Some other functions of nanosystems are to improve nutritional quality, flow properties, and stability, or increase the shelf life of otherwise degradable compounds as protection against oxidation, retention of volatile ingredients, moisture and/or pH-triggered, controlled release, consecutive delivery of multiple active ingredients, long-lasting organoleptic perception, enhanced bioavailability of essential nutrients, and improve rheological properties [15]. All these properties help to develop better food for consumers. The sensorial properties may or not change, according to the purpose, for example, it has been shown that nanoemulsion does not change the appearance of the food when added, but in other cases, it can be used for delivery of lipophilic ingredients such as flavor components, then they can mask unpleasant tastes and odors, as in fish oils. They can also help reduce calories by increasing the water and/or air content as in nanoemulsions, such as ice cream, reducing sugar and oil content by decreasing the size of air bubbles, and thus increasing creaminess [16]. For example, the use of nanoemulsions with *Zataria multiflora* and cinnamaldehyde essential oils for the preservation of ground beef patties cooked in a microwave showed better sensorial properties, improving this characteristic [17]. Thus, according to nanosystems applied in food, the sensorial properties can be modified, preferably to improve these characteristics.

## 4. Nanosystems in Food and Meat Preservation

Table 1 shows the principal nanosystems used in food processing, preservation, new product development, packaging, nutraceuticals, coatings, and applications designed to ensure alimentary security and extend shelf life. Today, the tendency is to use eco-friendly methods to prepare nanosystems and obtain more functional, stable systems with the capacity for the controlled release of natural additives during processing, storage, and/or distribution.

Nanosystems can be elaborated on using organic and/or inorganic materials. The latter include Ag, ZnO, TiO_2_, SiO_2_, SiO_3_, and CuO. These compounds are added to packaging materials to improve mechanical and thermal resistance and antimicrobial activity. None of these inorganic components are approved as ingredients in food. Inorganic nanosystems can include transition metals and metal oxides (silver, gold, iron, titanium, zinc oxide (ZnO), silica, titanium dioxide (TiO_2_), and iron oxides), alkaline earth metals (calcium, magnesium), and non-metals (selenium, silicates). These enhanced packaging materials offer superior food protection against external mechanical, thermal, chemical, or microbiological effects by providing an additional level of safety and functionality. Inorganic nanosystems also improve barrier properties to gases and moisture, thermal resistance, and machinability. Nanosystems are normally used in the fabrication of packaging through mixing with polymeric materials, or as thin amorphous film, of 50 nm or less [32]. Preparation methods of metal oxide nanoparticles (NPs) include chemical means, biosynthesis, sol–gel, co-precipitation, electrochemical, wet chemical, pyrolytic, microwave, hydrothermal, and sonochemical processes, as well as psychosynthesis. Very few inorganic–organic nanosystems can be used as ingredients in meat and meat products. Most are utilized in the manufacturing of packaging that is in contact with food. One example is using ZnO nanoparticles as an antimicrobial agent and nanosensor that can detect food degradation during packaging [33,34]. In meat packaging, ZnO nanoparticles effectively control both Gram-positive and Gram-negative bacteria, fungi, algae, and viruses related to electrical and catalytic properties and thermal stability [35]. These nanoparticles have multifunctional effects: high antimicrobial efficacy, piezoelectricity, optical transparency, and electrical conductivity. They also provide UV protection [36]. ZnO nanoparticles are used in active packaging as an antimicrobial agent since they help maintain meat quality, especially by controlling color and fat oxidation. They may affect flavor, but are considered safe by the FDA [37]. Copper nanoparticles are of interest due to the low cost of the materials required, negligible sensitivity to human tissues, and high sensitivity to microorganisms. Copper ions, meanwhile, function as a liquid sterilizer and an antibacterial, antifungal, antiviral, and antifouling agent that is effective against bacteria, fungi, algae, and viruses [38].

The aim of combining distinct organic-inorganic or organic–organic materials in the elaboration of nanosystems is to modify their functionality, perhaps to increase thermal resistance, adjust bioactive release, enhance processes and storage, or obtain better bioactive availability in the human organism. Mixtures of organic-inorganic compounds can improve compatibility because greater solubility increases interaction with key components of meat and meat products [39]. For example, ZnO nanoparticles are used with calcium alginate to combat *S. aureus* and *S. typhimurium* by reducing their presence in chicken after 10 days of incubation.

The so-called “green” metallic nanoparticle preparation methods offer ways to reduce pollution and help minimize undesirable effects of these NP [40,41]. Dissolvents such as ethanol, hexane, toluene, DMF (N, N-dimethylformamide), and diethyl ether have harmful safety, health, and environmental effects [42]. Table 2 shows nanoparticle preparation methods that utilize only environmentally friendly solvents. These solvents and surfactants are non-toxic and do not represent any risk for the environment. Moreover, they rarely lose their effectiveness, are preferably non-flammable, and can be recovered and reused [42,43].

## 5. Nanosystems and the Preservation of Meat and Meat Products

The meat industry constantly seeks new natural preservation methods to satisfy consumer demand, increase shelf life, and ensure the freshness, tenderness, and juiciness of meat while complying with all health and safety parameters. Nanotechnology is an approach that shows significant potential in this regard. Applying nanoparticles is one way of maintaining the physicochemical properties and microbiological quality of meat and meat products, but they can also reduce the use of ingredients such as salt, sugar, and other preservatives to improve bioavailability, intensify sensory properties, enhance the controlled release of active substances, and provide greater stability and absorption of active compounds with increased antimicrobial and antioxidant effectiveness [39]. Finally, nanotechnology can help identify adulterated meat and guarantee the authenticity of meat and meat products [14].

**Figure 2:**
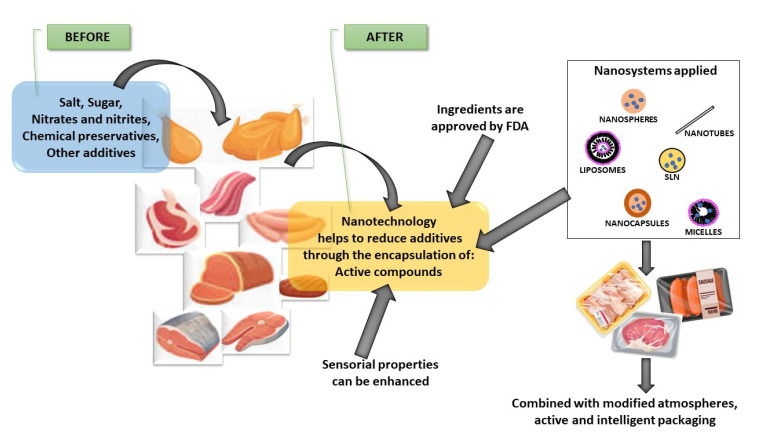
Technologies to improve the addition of additives in meat and meat products.

Food processing is important because it requires novel approaches to product development to obtain better products and increased shelf life as well as for the ease of handling products and easy preparation. In this sense, nanotechnology was integrated with traditional technologies as commercially successful foodstuffs added value to the final product. When the formulation is carried out, processing may involve heat/mass transfer operations, nanoscale reaction engineering and nanobiotechnology, and molecular synthesis which need to consider various aspects related to preserving the functionality at the nanoscale [15]. In Figure 2 show different nanosystems, additives can be added to reformulate and decrease the quantity of chemical ingredients, and they can be applied during animal feeding, processing, and packaging, and even in combining technologies, for example, with modified atmosphere and active and intelligent packaging, without forgetting the cost and capacity of the technology [18].

Another important point is that the existence of the COVID-19 makes consumers more worried about their health and well-being, and recommendations by international organizations are forcing meat industries to develop healthier products. For example, adding natural ingredients such as mushrooms (Cantharellus cibarius) were aggregated as antioxidants and antimicrobials in cooked sausages, thus obtaining good results but changing the odor and taste of the sausages. Ethanol extract of mesquite leaf was added to pork patties to prevent oxidation with good results as a natural antioxidant. For these reasons, it is important to develop technologies for better feeding with good quality and sensorial properties [52].

### 5.1. Effect of Nanosystems on Lipid Oxidation Control

During the post-mortem period and storage, meat and meat products deteriorate principally due to proteolysis and lipid oxidation. The oxidation of polyunsaturated fatty acids is responsible for rancidity, the development of off-flavors, the formation of toxic compounds, and nutrient and drip loss, but also affects color, nutritional value, texture, and consumer acceptance, and reduces shelf life [53]. Generally, lipid rancidity is determined by measuring peroxide (PV) and thiobarbituric acid reactive substance (TBARS) values. Adding natural antioxidants, such as essential oils and plant extracts, is one option for delaying lipid oxidation in meat and meat products. These substances can also enhance flavor and are classified as Generally Recognized as Safe (GRAS) [54]. However, one problem with natural antioxidants is their high sensitivity to light, pH, temperature, and gas concentrations, among other ambient conditions. Nanosystems can protect, stabilize, and reduce these problems during the processing and storage of fresh meat and meat products. The beneficial properties of nanometric-size coatings include increasing the contact area enhancing functionality and the capacity to release a natural additive at a precise site and time. They can be applied directly to meat products or be used to supplement animals’ diets [55,56]. Table 3 summarized some key studies on the application of nanosystems to prevent oxidation in meat.

Essential oils from oregano, rosemary, thyme, cinnamon, pepper, sage, basil, turmeric, ginger, garlic, nutmeg, clove, fennel, and coriander, among others, are natural antioxidants [55,56,66], that can be used alone or combined to extend shelf life [67]. For example, chitosan NP loaded with cinnamon essential oil effectively retard lipid oxidation in beef patties during storage at 4 °C for 8 days when added to the formulation of the meat [3]. Baldissera et al. (2020) [61] found that Nile tilapia fish fed a dietary supplement with Ne NS showed significantly reduced oxidation.

Plant extracts are rich in phenolic compounds, including tocopherols, flavonoids (flavones, flavanones, flavan-3-ol, and anthocyanins), phenolic acids, and terpenoids such as limonene, α-pinene, and camphor. These compounds are structurally related but differ in quantity and type depending on their source [68,69]. Carotenoids (β-carotene, lycopene), hydroxycinnamic acids (para-hydroxycinnamic acid), and antioxidant vitamins (ascorbic acid, dL-α-tocopherol) have also been used [70]. Elbarbary et al. (2015) [57] developed chitosan–vitamin C nanoparticles (CS–VC NP) with a size range of 23–82 nm. They reported that dipping fresh ground meat in this nanosystem had an excellent effect on delaying peroxidation by extending the induction period thanks to its antioxidant properties. Wang et al. (2020) [62] found the same effect when they applied eugenol nanocapsules embedded with gelatin–chitosan to pork (stored at 4 °C for 15 days). The treated meat had a lower rate of increase in TBARS than control samples. A fat-soluble free radical scavenger, the eugenol-mediated hydroperoxide protected muscles from oxidation and acted as an effective natural antioxidant that inhibited lipid oxidation. Alirezalu et al. (2021) [71] added ε-polylysine NP (ε-PLN) combined with plant extracts (green tea, olive leaves, and stinging nettle) as a nitrite replacer in frankfurter-type sausages. They determined that the sausages formulated with the ε-PLN had significantly higher contents of phenolic compounds and lower TBARS values after 45 days of storage at 4 °C.

Some fruit extracts are also good sources of antioxidants and have been applied at the industrial level with great acceptance. Extracts of apples, blueberries, plums, grapes, black currants, and pomegranates contain relatively high concentrations of flavonoids, while grape seed proanthocyanidin extract provides significantly higher protection against free radicals [68,72]. After adding lyophilized pomegranate peel NP (LPP NP) to a formulation of meatballs, Morsy et al. (2018) [59] found that TBARS content was lower than in the controls after 15 days. LPP NP antioxidant activity is attributed mainly to compounds such as phenolic hydroxyl groups and double bonds that include hydrolysable tannins and flavonoids. They also reported that this activity is due to a multi-factorial impact and the synergistic chemical action of numerous compounds. Ibrahim et al. (2021) [65] developed a dietary treatment formulated with quercetin nanoparticles (QT NP) for Nile tilapia fish. Quercetin is the main polyphenolic flavonoid found in various fruits and vegetables. It acts as a potent antioxidant in biological systems to protect tissues from free radicals. Those authors reported that ROS production and malondialdehyde (MDA) content in fish muscle decreased markedly with increasing dietary QT NP supplement levels, suggesting a reduction in free radical content and less lipid damage, possibly because administering a QT NP-enriched diet decreased ROS production by increasing cellular resistance to oxidative stress with a subsequent decrease in lipid peroxidation and higher numbers of healthy cells.

Non-loaded chitosan NP are another natural alternative for preventing lipid peroxidation in meat at a particle size ranging from 1–1000 nm [73]. They have shown significant antioxidant properties involving the formation of a fluorophore through interaction with chitosan and malondialdehyde amino groups, the initial compound formed during oxidation of polyunsaturated fatty acids [74]. Bonilla et al. (2012) [75] sustained that chitosan may chelate the free iron released during meat storage from myoglobin during spoilage. This would explain its antioxidant effect. In addition, free amino chitosan groups can scavenge free radicals to create stable radicals and hydrogen ions and form ammonium (NH_3_^+^). In this regard, developing and applying solutions of chitosan-sodium tripolyphosphate nanoparticles (CS–TPP NP) to shrimp by vacuum tumbling was studied. In that work, quality characteristics were evaluated during 120 days of frozen storage (−20 °C). The CH–TPP NP treatments generated the highest reduction in lipid oxidation compared to other treatments in this period as those samples had the lowest TBARS value [58]. Finally, Hajji et al. (2019) [60] studied the effect of adding CS–TPP NP to a surimi formulation. They found that the system exhibited an inhibitory role against lipid oxidation by reducing TBARS values and the formation of conjugated dienes during 9 days of storage at 4 °C.

### 5.2. Application of Nanosystems to Control Microbial Growth in Meat

Meat is a product with high water content that presents variations in pH during storage and is susceptible to microbial growth. The main microorganisms present in fresh meat are bacteria, but yeast and mold can develop in some products, resulting in contamination that can decrease shelf life and, more seriously, cause foodborne diseases. The exact types of microorganisms that affect meat depend on the physiological condition of the animal pre-slaughter, storage conditions, handling and manufacturing practices, and the initial microbial load. Raw meat is more prone to microbial growth because of the microorganisms that exist on its surface [76]. The main bacteria that affect meat and meat products include *Acinetobacter*, *Brochothrix thermosphacta*, *Enterobacter*, *Lactobacillus sakei* and *curvatus*, and *Pseudomonas*. Yeasts and molds can cause defects such as off-staste and off-odors. Other pathogenic microorganisms found on meat are *Aeromonas hydrophila*, *Campylobacter jejuni*, *C. perfringens*, *E. coli* O157: H7, *L. monocytogenes*, *S. enterica*, *S. typhimurium*, *S. aureus, B. cereus* and *C. botulinum* spores. These agents are not necessarily eliminated permanently by heat treatments, so for decades several synthetic agents have been used to prevent their growth (e.g., benzoic and sorbic acid and derivates, paraben, sulfites). However, toxicological problems have been linked to these additives, leading to the search for natural alternatives that prolong shelf life without causing problems of this kind [52].

Research shows that the synthesis of nanoparticles made from, or loaded with, organic or inorganic antimicrobial compounds improves the characteristics and efficiency of those compounds by increasing their surface to allow greater contact with microorganisms. The antimicrobial effect of nanosystems depends on the type of nanoparticle used and its size, shape, and surface charge [39]. In relation to microorganisms, the nanoparticle effectiveness depends on the structure of the wall of bacterial cells (outer layer charge, hydrophobicity, or hydrophilicity), their growth rates (slow-growing bacteria are more resistant to nanoparticles than fast-growing strains due to their expression of stress response genes), and their potential for film formation (the ability of pathogenic bacteria to form biofilms protects them against antimicrobials). The action mechanism of antimicrobial nanoparticles is still under study, but some authors report that nanoparticles damage the membrane surface of microorganisms through protein binding, enzyme and nucleic acid disruption, and/or by interfering with various metabolic processes [59,77]. Important organic antimicrobials that have been extensively studied include chitosan, essential oils, plant extracts, and microbial colorants. Table 4 lists key studies on the use of nanosystems, most of them loaded with bioactive compounds, and their inhibitory effects on various microorganisms.

Essential oils are natural antimicrobials whose activity improves when they are applied in nanosystems that enhance their chemical stability and solubility, minimize evaporation and degradation of their active components, and present a controlled, sustained release. The antimicrobial effect of essential oils is due to bioactive compounds such as terpenes, aliphatic alcohols, aldehydes, and isoflavonoids [8,9,40]. Ghaderi-Ghahfarokhi et al. (2017) [3] reported a positive effect of chitosan nanoparticles made of cinnamon essential oil (CEO–CS NP) on inhibiting *S. aureus* on beef patties stored at 4 °C, compared to control samples during 8 days of storage. The growth of *S. aureus* was 3.99 and 4.59 CFU/g in samples treated with 0.05 and 0.1% of CEO–CS NP, compared to control samples that reached 6.85 CFU/g in the same interval. Hadian et al. (2017) [78] studied the antimicrobial effect of chitosan-benzoic acid nanogels loaded with *Rosmarinus officinalis* essential oil (REO–CS-BA NG) against *S. typhimurium*. They applied the nanosystem by spraying it on the meat surface, then storing the samples at 4 °C for 12 days. They found that adding 1 mL of nanocapsules of REO (at various entrapped concentrations) caused an immediate reduction in *S. typhimurium* populations from day 1. On day 12 of storage, the bacterial population on the treated samples was significantly lower than on the control. In their work, Moraes-Lovison et al. (2017) [79] developed a nanoemulsion that encapsulated oregano essential oil (OEO NE). Results showed that the OEO NE had an antibacterial effect on *E. coli* and *S. aureus* when added to a formulation of chicken pâté. Rajaei et al. (2017) [67] observed that applying chitosan–myristic acid nanogels loaded with clove essential oil (CEO–CS-MA NG) to the surface of beef cutlets stored at 4 °C for 12 days markedly reduced the growth of *Salmonella* (*p* < 0.05). Collectively, these results demonstrate that the encapsulation of bioactive compounds can enhance the antimicrobial activity of essential oils by increasing their dispersion in aqueous environments and stimulating passive adsorption mechanisms into cells.

Several plant extracts have also been shown to have antimicrobial effects due to the presence of phenolic compounds such as phenolic acid, flavonoids, and tannins. Their efficacy generally depends on the concentration and chemical structure of the bioactive agent. Plant essential oils tend to be more effective on Gram-positive than Gram-negative bacteria, perhaps due to the lipopolysaccharide outer membrane that surrounds the cell wall of the latter type [80]. Wang et al. (2020) [62] reported the lowest total bacteria count (TBC) on pork treated by dipping in eugenol gelatin chitosan nanoparticles (Eug–Gel-CS NP). They also observed that their nanoparticles exerted a sustained release effect of Eug that could prolong its antiseptic and antibacterial effects. Meat products such as sausages have also been treated with nanoparticles to improve shelf life. Alirezalu et al. (2021) [63] found that formulating frankfurter-type sausages with ε-polylysine (ε-PLN) significantly increased shelf life when stored at 4 °C for 45 days due to ε-PLN’s efficient inhibition of total viable count microorganisms such as *Clostridium perfringens, Staphylococcus aureus*, and *E. coli.* Tometri et al. (2020) [81] assessed the microbial effect of bay leaf encapsulated in nanoliposomes on meat sprayed with 1000 and 1500 ppm of the nano-extract and stored at 4 °C for 12 days. They reported that the highest values for *S. aureus* were observed in the control treatment on most days, while in the treatments containing the nanoencapsulated extract, no bacterial *S. aureus* was observed in the samples treated with 1500 ppm of the nano-extract on day 4 of storage. The authors concluded that higher concentrations of the extract had better results on inhibiting *E. coli*, and that the effect improved with the treatments that contained the nanoencapsulated extract. In fact, from day 8, no *E. coli* was observed in the samples treated with the encapsulated extract at 1500 ppm.

The use of geraniol as an antimicrobial increased effectiveness when added in liposomal nano-formulation obtained by microfluidics into the pig gastrointestinal tract and was shown to reduce the growth of *S. typhimurium*, thereby increasing the safety of the food supply chain [10].

**Table 4:** Summary of nanosystems applied on meat and meat products and their antimicrobial effect.

Nanosystem	Size (nm)	Applications and Effects	Product	Ref.
ZnO nanoparticle suspension containing acetic acid ZnO–AA NP	20–25	The meat samples were inoculated with *L. monocytogenes*, *E. coli*, and *S. aureus* (7 log CFU/g) and treated with various concentrations of ZnO NP suspensions (6 mM and 8 mM) plus 1% acetic acid by meat surface spraying and stored at refrigeration temperature. Higher concentrations of ZnO–AA NP significantly decreased the population of all microorganisms compared with the control. Populations of *L. monocytogenes*, *E. coli*, and *S. aureus* in meat decreased by 4.09 to 4.72, 0.84 to 1.24, and 2.12 to 2.75 log CFU/g, suggesting that treatments of ZnO–AA NP suspensions containing acetic acid can reduce the growth of the studied bacteria in meat.	Sheep meat	[82]
Chitosan–sodium tripolyphosphate nanoparticles CS–TPP NP	-	The authors reported that aerobic plate counts of chitosan nanoparticle treated shrimp were lower compared to other treatments during the entire storage time. CS–TPP NP have a greater surface area per unit volume and higher charge density than CS. Both of these factors greatly contribute to their interaction with anionic bacterial cell membrane. Fresh white shrimp meat was vacuum-tumbled with treatment solution (CS–TPP NP) and stored at 20 °C for 120 days.	Shrimp	[58]
Cinnamon essential oil-chitosan nanoparticles CEO–CS NP	235.6	According to the authors, no significant differences were observed among the treatments in the beginning of storage, but encapsulated CEO showed a distinguished inhibition pattern of *S. aureus* in the following days and at the end of the storage where the control samples reached 6.85 log CFU/g while meat treated with nanoparticles reached 3.99 and 4.59 CFU/g (0.05 and 0.1% of encapsulated CEO). The use of CS NP as a carrier of CEO looks promising, as lower microbial growth was obtained. The nanoparticles were added to the meat as part of the formulation, samples were stored at 4 °C for 8 days.	Beef Patties	[3]
Chitosan–benzoic acid nanogels loaded with *Rosmarinus officinalis essential oil* REO–CS-BA NG	<100	The antimicrobial effect of REO–CS-BA NG against *S. typhimurium* on beef cutlet samples was evaluated in vivo during 12 days of refrigerated storage at 4 °C. 1 mL of the NC REOs (at various entrapped REOs concentrations) was spayed over the surface of the meat batches. REO–CS-BA NG caused an immediate reduction in *S. typhimurium* population since day 1. Moreover, after 12 days of storage, the population of the bacteria on the coated samples was significantly lower than that of the control.	Beef cuttles	[78]
Nanoemulsion encapsulating *oregano essential oil* OEO NE	35–55	Regarding contamination of chicken pâté by *E. coli*, on the last day of analysis, the OEO NE antibacterial effect was significantly higher (*p* < 0.05) than the other treatments. For *S. aureus*, the growth inhibition effects were similar for both pure and nanoencapsulated oregano EO, whereas treatment with nitrite (synthetic food preservative) was the most efficient in reducing the bacterial growth. The nanoemulsion was added to the chicken pâté as part of the formulation.	Chicken pâté	[79]
*Clove essential oils* encapsulated by chitosan myristic acid nanogel CEO–CS-MA NG	<100	The surface treatment of beef with 1 and 2 mg CEO–CS-MA NG resulted in significant reductions in *Salmonella* populations by 0.58 and 0.68 log CFU/g, respectively, compared with the positive control after 3 d of storage. These results show that encapsulation of bioactive compounds may theoretically enhance the antimicrobial activity of essential oils, by increasing their dispersion in aqueous environment and stimulating passive adsorption mechanisms into cells. Beef cuttles were stored at 4 °C for 12 days.	Beef cuttles	[67]
Lysozyme, nisin, EDTA, ZnO NP (LNEZ)	<110	Antimicrobial solution was directly applied on the meat surface after attachment and spread uniformly, meat was packaged and stored at 4 °C for 15 days. Treated and control samples were inoculated with 7 log CFU/cm^2^ of *E. coli*, *L. monocytogenes* and *B. cereus*. The application of LNEZ against *E. coli* O157:H7 showed a reduction (2.7 log CFU/cm^2^) in these bacteria after 15 days of storage, while control samples reached a population of 8.1 log CFU/cm^2^ over the course of the experiment. Same results were observed for *L. monocytogenes* and *B. cereus*, showing a final concentration of 2 and 4 log CFU/cm^2^ after 15 days at refrigerated conditions while control samples reached more than 8 log CFU/cm^2^, respectively.	Minced beef	[59]
Bay leaf encapsulated nanoliposomes BL NL	99	The meat was sprayed with 1000 and 1500 ppm of nano-extract and stored at 4 °C for 12 days. For *Staphylococcus aureus*, the highest values were observed in control treatment on most days while in the treatments containing the nanoencapsulated extract, no bacterial *S. aureus* was observed in at 1500 ppm non-extract on day 4 of storage. *E. coli*, higher concentrations of the extract showed better results and also the results were better with the treatments containing the NC extract so that from day 8 no *E. coli* were observed in the NC extract at 1500 ppm.	Minced beef	[81]
Eugenol nanocapsules embedded with gelatin –chitosan Eug–Gel-CS NP	229.09 nm	According to the results, the total bacteria count (TBC) of the meat samples in each treatment group increased as the storage period was extended. The Eug–Gel-CS NP group exhibited the lowest TBC growth rate. The Eug nanoparticles were verified to exert a sustained release effect on Eug, which could prolong its antiseptic and antibacterial effects. Meat was immersed in Eug–Gel-CS NCs formulation and stored for 15 days at 4 °C.	Chilled pork meat	[62]
ε-polylysine nanoparticles with plant extracts ε-PLN	-	Sausages formulated with ε-PLN significantly increased shelf life of frankfurter-type sausages stored at 4 °C for 45 days, since values of total viable count microorganisms did not increase significantly during storage, reaching to 4.03 Log CFU/g after 30 days. Regarding *Clostridium perfringens*, the percentage of decrease was significantly higher in ɛ-PLN samples than in the other groups, not being detected at the end of storage. The same behavior was observed in *Staphylococcus aureus*, one of the most important bacteria in meat and meat products due to production of enterotoxins. ε-PLN were efficient against *E. coli* compared to the other groups. After 45 days of storage ɛ-PLN sausages reached to not detected values, while control samples displayed values of 0.21 Log CFU/g.	Frankfurter-type sausages	[63]

Another approach often adopted in meat preservation to minimize microbial resistance involves inorganic nanoparticles. The advantages of these antibacterial reagents (e.g., metal oxide) over organic materials are low biotoxicity and greater stability at high temperatures and pressures [83]. Materials commonly used to produce inorganic nanoparticles include silver, titanium, gold, and zinc, which are four oxide metals. Of the four, ZnO offers the greatest advantages because it has the highest photocatalytic efficiency, is more biocompatible, and has better selectivity, durability, and heat resistance. It can be used to combat diverse microorganisms, including *S. aureus*, *E. coli*, and *C. albicans*. Decreasing the particle size of this antimicrobial agent produces a quantum confinement surface with changes in electrical, optical, and chemical properties, while intensifying its activity against microorganisms [84]. Since nanoparticles act in a non-specific manner, multiple mechanisms may underlie their activity, such as (i) the production of reactive oxygen species (ROS); (ii) the loss of cellular integrity following contact between ZnO NP and the cell wall; (iii) the release of Zn^2+^ ions; and (iv) ZnO NP internalization. Table 4 shows some studies that elucidate the preservative effect of applying inorganic nanoparticles (ZnO) on meat or meat products.

**Figure 3:**
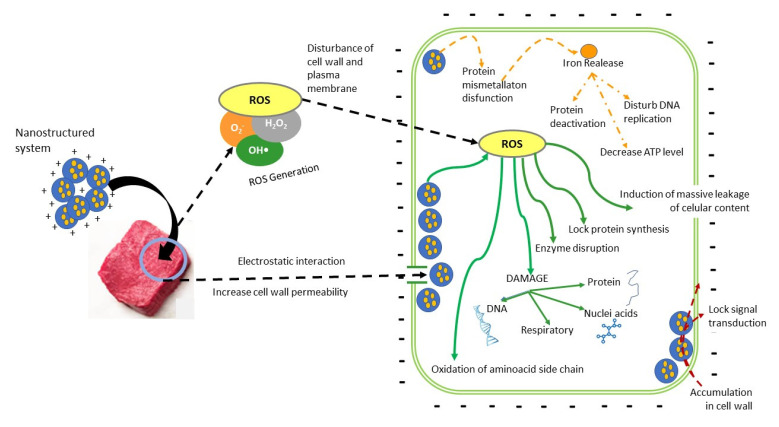
Action mechanism of nanostructured systems as antimicrobial agents applied to meat.

Mirhosseini and Arjhmad (2014) [82], for example, studied the effect of spraying a ZnO nanoparticle suspension on meat. Samples were inoculated with *L. monocytogenes*, *E. coli*, and *S. aureus* (7 log CFU/g) and then treated with two concentrations of ZnO NP suspensions (6 and 8 mM) plus 1% acetic acid (AA) and stored at refrigerated temperature. The higher concentration of ZnO–AA NPs significantly decreased the populations of all microorganisms compared to the control. Populations of *L. monocytogenes*, *E. coli,* and *S. aureus* in the meat suggesting that treatment with ZnO–AA NP suspensions containing AA can reduce the growth of these bacteria in mutton. Morsy et al. (2018) [59] analyzed the synergistic effect of several antimicrobials, including ZnO nanoparticles, on the inhibition of *E. coli*, *L. monocytogenes*, and *B. cereus*. Their work demonstrated that applying lysozyme-nisin-EDTA–ZnO NP (LNZE) on the meat surface had a powerful effect on reducing these bacteria (2.7, 2, and 4 log CFU/cm^2^. respectively) after 15 days of storage at 4 °C, while all control samples reached levels >8 log CFU/cm^2^. Figure 3 outlines the action mechanisms of nanosystems on microorganisms in meat products. Pateiro et al. (2021) [52] mention that the antimicrobial activity associated with nanoemulsions is linked to the small particle size (in most cases <100 nm), which confers high stability. Their action occurs when the active compounds in the tiny droplets penetrate the bacterial membrane, causing the release of the cytoplasmic contents. The inhibitory effect of metal oxide nanoparticles (MONP) depends on their concentration, size, and form [85]. Ali et al.(2021) [77] explain that the generating ROS (O_2_^−^, OH•, H_2_O_2_) is the main antimicrobial action of MONPs, which mainly inhibits respiratory enzymes. Bacterial membranes are negatively charged due to the presence of carboxylic acid groups on proteins, while MONPs are positively charged. This difference in charge between the bacterial membrane and the MONPs produces electrostatic attraction that allows the MONP to accumulate on the cell surface until they can penetrate the bacteria [32]. Ali et al. (2021) [77] also mention that MONPs alter the structure and permeability of the cell membrane and attract more MOPNs into the cytoplasm with simultaneous leakage of cellular content, producing toxicity, nucleic acid damage, and photokilling. Metal ions (positively charged) bind to DNA, alter the helical nature of strands, and interact with single-bonded SH groups in the peptidoglycan layer, causing destruction of the cell wall. When MONPs bind to mesosomes, they can also disrupt cellular respiration, cell division, and DNA replication [32,77].

### 5.3. Application of Nanosystems in Meat Processing

The industrial processing of meat products requires additives that accelerate processes, stabilize color, and improve flavor. Nanosystems can reduce the fat levels of products by interacting with components in meat, and preserve flavor [86], minimizing the levels of salts such as nitrates, sodium, and phosphates and the incorporation of prebiotics, probiotics, and other functional ingredients for the development of healthier meat products [14]. The color of meat and meat products is one of the most important parameters for consumers, and is related to the freshness and palatability of fresh and processed products [87]. While consumers can judge the color of meat at purchase, they cannot appreciate its flavor, juiciness, and tenderness until it is cooked. Myoglobin is the sarcoplasmic protein responsible for color, though hemoglobin and cytochrome C also participate in beef, lamb, pork, and poultry [88]. Color changes depend on the oxidation or reduction in myoglobin due to endogenous factors such as pH, the source muscle, antioxidants, lipid oxidation, and mitochondrial activity [87]. Other factors that can affect color are handling, diet, age at slaughter, pH, and genetics. Exogenous factors include oxygen, temperature, light, and the meat’s initial microbiological load [89].

It is important that meats such as beef do not lose their characteristic red color during storage, so is crucial to develop technologies that preserve and/or enhance this property. Two methods often used are nanoparticles and modified atmospheres. When used with fresh meat, the latter contain around 80% O_2_ and 20% CO_2_. Nano-encapsulated natural antioxidants, meanwhile, have been shown to improve the color stability and shelf life of both whole muscle cuts and ground meats [89].

Nanosystems can play an important role in protecting functional ingredients from chemical or biological degradation (e.g., oxidation) during processing, storage, and use, and can control the release of active agents or the conditions that trigger their release (e.g., pH, ionic strength, storage and processing temperatures) [66,90]. For example, in the industrial production of sausages and cured meats, numerous additives are required to accelerate processes and for new formulations that minimize or modify fat content and reduce sodium, phosphate, and nitrate content. New formulations may incorporate probiotics, prebiotics, other materials, such as algae and nuts [39], or encapsulated active ingredients such as vitamins C and E and fatty acids that can be used as preservatives and processing aids. These advances can be achieved using nanotechnology, always keeping in mind that they must be compatible with such attributes as the flavor, texture, and color of the final products [91].

One significant discovery during research on these kinds of applications is that ginger in the form of micro-sized powders can be used as a tenderizer and extender that improves penetrability, solubility, and dispersibility. This suggests that certain ingredients can be reduced to nanopowders that offer new physical and chemical properties favorable for meat processing [39].

The application of nanoemulsions with chitosan have antimicrobial properties, because the amino groups which are positively charged and microorganisms are oppositely resulting in the outflow of proteinaceous and other intracellular constituents of the microorganisms, this structure entraps a wide array of lipophilic active ingredients, they can be applied to meat and meat products, for example, maintained the color of fresh pork when stored at 4 °C, in the beef loins prolonged shelf life [92]. Another application is the inclusion of meat-derived bioactive peptides, pro- and prebiotics in processed meat products, nanosensors and nanotracers for meat bio security tracing and nanostructured meat products with defined functions [91]. One of the newest applications, is where the fibrillar protein aggregates are developed as meat replacers, and nanotechnology may help to enable fibrillar proteins to be constructed to imitate meat [93]. It is also possible to incorporate chromium nanoparticles in the feed of pork and chicken resulting in meat with better quality, increase in skeletal muscle mass in pork, and in chicken positively affected breast and thigh muscle protein content while simultaneously lowering cholesterol, obtaining better characteristics for the consumers [94].

## 6. Application Methods

### 6.1. Marinating

Marinating is the most common method used in meat preservation. It consists of placing meat or meat products in a liquid solution marinade to improve their flavor, texture, and juiciness. Marinades may also contain ingredients that enhance product appearance, such as salt, organic acids, water, seasonings, spices, sugar, agglutinants, aroma enhancers, antimicrobial, or antioxidant agents (which can be nanoencapsulated to intensify their effect), and colorants. Some of these ingredients can be nanoencapsulated to give the marinated product a suitable color. The selection of ingredients for marinade formulations, process control, and technological approaches is being refined continuously to improve product quality characteristics and consumer satisfaction [95].

### 6.2. Edible Coatings

Edible film materials can be made of proteins, polysaccharides, or lipids, among other substances, to ensure that they are easily degradable after they fulfill their function [80]. Since edible antimicrobial films tend to have high moisture sensitivity, a poor water barrier function, and better mechanical properties than synthetic polymers, plasticizers are often blended with biopolymers to improve processing, increase flexibility, and decrease the glass transition temperature. The plasticizers most often used are glycerol, sorbitol, mannitol, fructose, mannose, and polyethylene glycol. Because polysaccharide-based films have low gas permeability, they extend the shelf life of food products without creating anaerobic conditions. However, the hydrophilic groups in their structure make them sensitive to moisture. Commonly used polysaccharides include starch, alginate, cellulose, chitosan, pectin, and carrageenan. Protein-based edible films have lower water resistance and less mechanical strength than synthetic forms, but proteins generally exhibit a greater ability to form films with better mechanical and barrier properties than polysaccharides. Common proteins used come from whey, soy, corn zein, and/or their derivatives [9].

The properties of these edible films depend on the characteristics of the nanomaterials used to form their layers. Layers made of lipids act as moisture barriers but are not efficient in blocking gases and have low mechanical resistance. Protein- and polysaccharide-based coatings, in contrast, form an effective barrier against gases but not moisture, so they can be used as natural, edible barriers that extend shelf life and provide nutritional properties [39].

### 6.3. Processing Ingredients

When meat is processed, biochemical changes such as proteolysis, lipolysis, and oxidation occur, so meat products develop typical volatile aromatic compounds during, for example, curing and fermentation. While oxidation is the leading cause of the deterioration of meat quality during processing and storage, it is also a crucial reaction for developing the typical flavor of meat products, especially in the case of dry-cured products that undergo a long maturation process. Vegetable proteins, dietary fibers, herbs, spices, and probiotics have all been used to reduce product costs and improve functionality [6,14]. This suggests that nanosystems could also be employed by encapsulating active ingredients that are effective during processing. For example, in one study incorporating oregano (*Origanum vulgare*) essential oil into nanoemulsions decreased microbial growth and extended the shelf life of chicken pâté. A synergistic effect of cinnamon essential oil and other antimicrobials has also been observed in fresh pork and beef sausage [52].

## 7. Future Trends

Nanotechnology has had such a great impact that research is increasing on its applications in the food industry and, specifically, the processing of meat and meat products. The aim is to develop economical, high-performance, easy-to-adapt methods using novel ingredients that are not chemical-based and do not pollute the environment. Pickering nanoemulsions are a case in point. Since they do not contain surfactants (conventional chemical emulsifiers), they can be added to films and used in the packaging stage to improve water vapor barrier properties, transport hydrophobic active compounds, control the release of compounds, and extend activity time. Other active compounds that are gaining interest are polyphenols. In addition to acting as antioxidants, they act as crosslinking agents that improve physical and mechanical properties when added to films. Finally, biodegradable films developed from polysaccharides extracted from natural sources are widely accepted today because they do not pollute the environment.

The development of new methods for obtaining nanosystems is spurred not only by producers, industrialists, and researchers, but also by consumers who demand high-quality meat products, safe foods, and, above all, foods with specific nutrients that contribute to maintaining people’s health. These advantages, however, come with higher costs that consumers must be willing to pay for the added value of meat products. Their willingness to do so will lead to the development of a supply and demand system that will further advance this technology.

## 8. Conclusions

Currently, the meat industry seeks not only to better preserve meat and meat products, but also to improve quality and give added value by offering healthier products and reducing amounts of fat, cholesterol, sodium chloride, and nitrites, among other substances. This entails modifying formulations and integrating cutting-edge technologies that reduce the need for synthetic additives. One result is that nanotechnology is gaining wider acceptance and producers are willing to invest in developing nanostructured systems that improve their products and add value. These measures include increasing the bioavailability of nutrients and obtaining controlled release of active ingredients, so they function continuously during the processing, storage, and distribution of meat products. In addition, it is possible to improve systems for detecting chemical reactions in meat products that will increase their shelf life and marketing time while maintaining key physical, chemical, microbiological, and sensory characteristics.

Finally, we cannot lose sight of the legal aspects of the use of ingredients in the elaboration of nanoparticles, since all substances must be FDA-approved. Other key factors to be considered include eliminating the use of solvents that are harmful to the environment and developing methods that do not contaminate and can easily be integrated into the processing and packaging of meat and meat products. Although much research remains to be carried out, scientists and producers alike are interested in developing and investing in accessible, economical methodologies that will benefit their operations and consumers by delivering products with greater added value, excellent quality, and high nutritional value.

## Figures and Tables

**Table 1:** Nanosystems applied in food preservation.

Nanosystems	Types and Definitions	Application in Food	Ref.
**Polymeric nanoparticles** Nanosystems with a wall membrane or structure composed of natural or synthetic biodegradable polymers. All materials must be approved for use in food [18].	**Nanocapsules** are composed of a biopolymeric wall surrounding a core that contains an active compound [19].	Fresh cut apple, orange juice, tuna fish, lean beef, fresh cut melon	[12]
	**Nanospheres** are matrix systems that incorporate a bioactive compound entrapped and chemically-bound or absorbed into a polymer matrix. The bioactive compound disperses inside the nanosphere polymers [6].		
**Surfactant-based systems**	**Nanoliposomes** are lipid vesicles and consist of concentric bilayers, each with two layers of surfactant molecules whose non-polar tail groups face each other [20].	Dairy products	[21]
	**Micelles** are thermodynamically-stable structures typically made from surfactants with groups of polar heads and non-polar tails [22].	Bakery products	[23]
**Emulsion-based systems**	**Nanoemulsions:** a lipid phase is dispersed in an aqueous continuous phase or vice versa. They are thermodynamically-unstable and improved by incorporating stabilizers, emulsifiers, weighting agents, ripening inhibitors, and texture modifiers [24].	Cabbage, cucumber, grape berry, green beans, Orange juice	[4,25]
	**Solid lipid nanoparticles (SLN)** are nanoemulsions that contain active substances prepared with lipid solids at ambient temperature (high melting points). At cold temperatures, they solidify for use as delivery systems [26]. **Nanostructured lipid carriers (NLC)** are the next generation of lipid nanoparticles. They are prepared from a mixture of solid and liquid lipids to reduce the melting point [27].	Guava (*Psidium guajava* L.) Fortified Beverages	[4,28]
**Nanotubes**	**Nanotubes** are ultrathin tubes of nanometer-size diameter whose constituent elements are carbon, boron, and sand silicon that impart excellent thermal and electrical conductivity [14].	Carrot, apples	[4]
**Electrospinning**	**Nanofibers:** electrospinning is used to obtain nanofibers, which are produced using high voltage that generates a field on a jet of a polymer solution. The nanosystems obtained have a large area:volume ratio, high inter-intra pores, high encapsulation efficiency, and better control of release and protection of active compounds [29].	Food processing	[30]
**Nanolaminates**	**Nanolaminates** are considered to consist of two or more layers of material of nanometric dimensions that are bonded physically or chemically [20].	Nutraceutical food, pork preservation, fresh cut and whole pears and mangoes	[25]
**Nanohydrogels**	Networks of three-dimensional hydrophilic or amphiphilic polymers that can swell in water (to around 30 times their size) and contain a relatively large amount of aqueous solvent. They maintain their structure thanks to the presence of covalent or non-covalent interactions [24].	Enrich mayonnaise with fish oil	[31]

**Table 2:** Eco-friendly methods for obtaining nanosystems.

Method	Description
**IONIC GELATION** The *aqueous phase* contains a charged polymer and counterions such as calcium carbonate or calcium chloride. Phase A is compounded by a polymer added by dripping into Phase B, which contains the active agent, under constant stirring. Gelation is instantaneous when the two phases come into contact, forming a three-dimensional network. An example is using alginate–calcium chloride, which produces a structure called “egg box”, where the “eggs” are the calcium ions [8]. This technique does not require solvents or severe temperature and pH conditions.	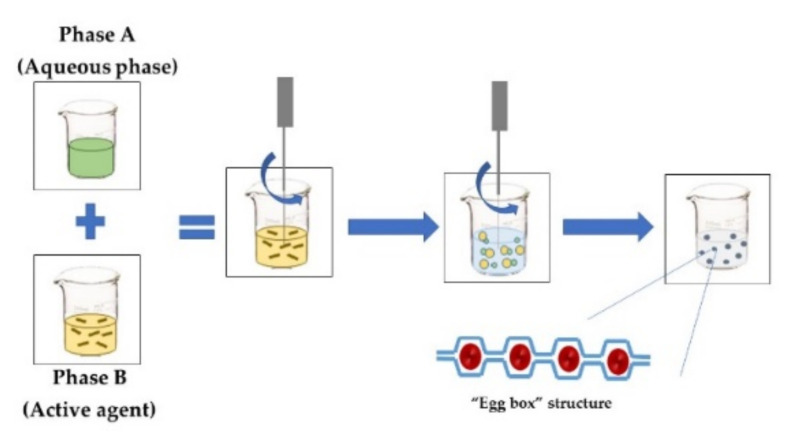
**MOLECULAR INCLUSION** This involves interactions between two compounds, a host molecule that contains the active compound in a cavity-bearing material, such as cyclodextrin [19], which has a hydroxyl group outside a doughnut-shaped molecule and a relatively non-polar hole in the center, where the active compound of appropriate size and polarity; the second element is placed to form “host–guest” inclusion complexes in an aqueous solution [44].	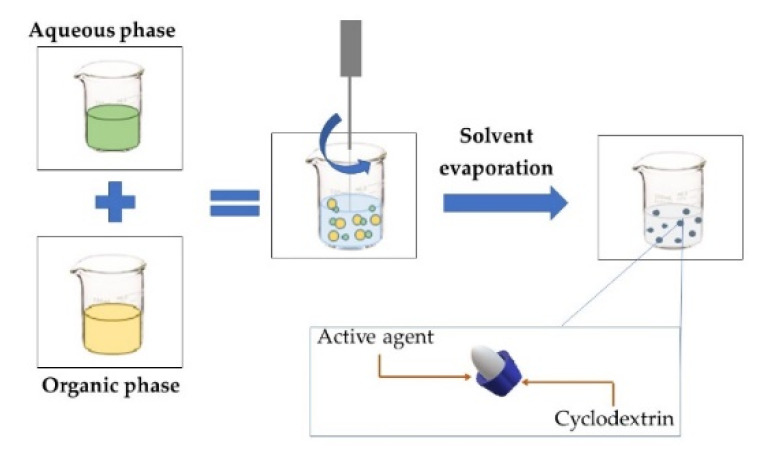
**NANOPRECIPITATION OR SOLVENT DISPLACEMENT** The *organic phase* contains an active agent and polymer that are dissolved in an organic miscible solvent (acetone, ethanol), and a protein or polymer that can be dissolved. The *aqueous phase* contains the stabilizer. The polymer precipitates, trapping the bioactive compound at the precise instant when the solvent containing the polymer diffuses into the dispersing medium [20,45].	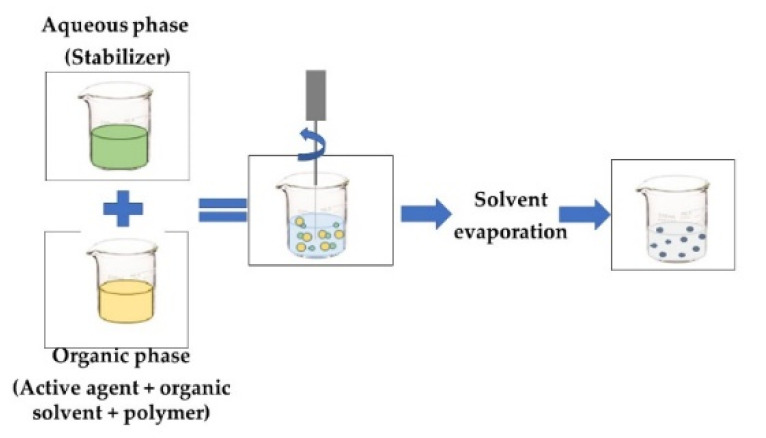
**EMULSIFICATION-EVAPORATION** The *organic phase* contains the active compound and a hydrophobic polymer. The *aqueous phase* contains a surfactant and polymers that form the emulsion. The o/w emulsion is homogenized by a high-shear device, such as a rotor-stator, pressure homogenizer, or ultrasound. The solvent is then removed to allow the active compound and polymer to precipitate [46].	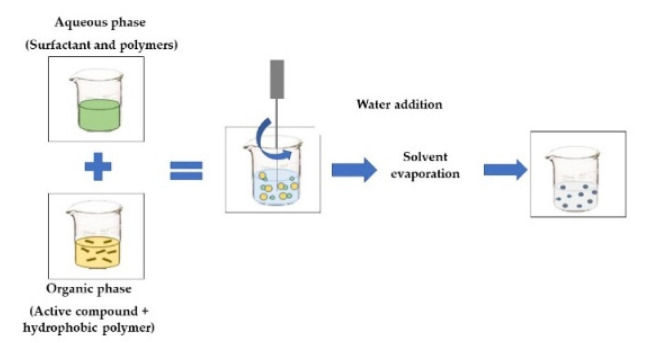
**SPRAY-DRYING** The sample is passed through a nozzle that forms a mist of fine droplets that atomize in a drying chamber and come into contact with hot air (temperatures of 100–200 °C) that causes spontaneous evaporation of the water. Once dried, the particles are passed through a cyclone or filter, where they are collected in powder form [47].	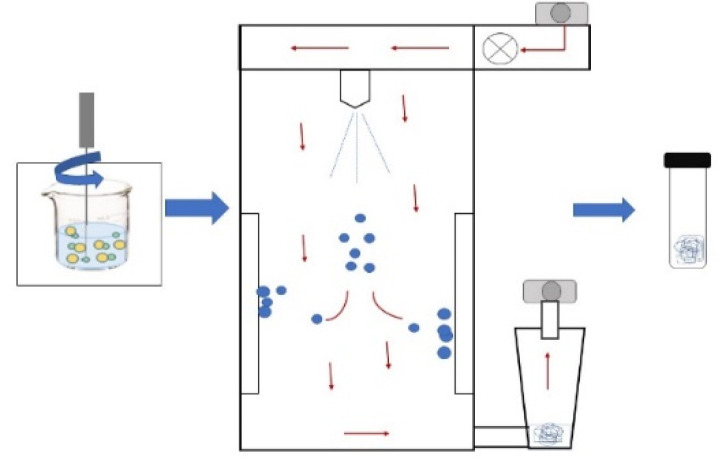
**LYOPHILIZATION** Nanoparticle dispersions are atomized in a stainless-steel spray tower lined with a liquid nitrogen cooling jacket so that the particles freeze as they fly in the cold air, avoiding contact with the liquid nitrogen. This is a 3-stage process: droplet formation, freezing, and freeze-drying [48].	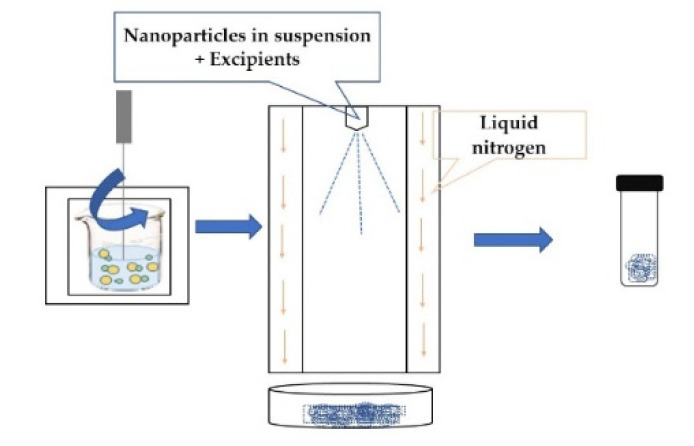
**SUPERCRITICAL FLUID EXTRACTION OF EMULSIONS** A supercritical fluid is used to rapidly extract the organic phase of a nanoemulsion, in which a bioactive compound and its coating polymer were previously dissolved. Upon removing the solvent, both compounds precipitate, generating a suspension of particles in water [49]. The supercritical fluid chosen should have high affinity for the organic solvent, but negligible affinity for the active compound. Due to the rapid supersaturation of the dissolution medium by the active compound, the latter precipitates on a nanometric scale, encapsulated in the surfactant material [50].	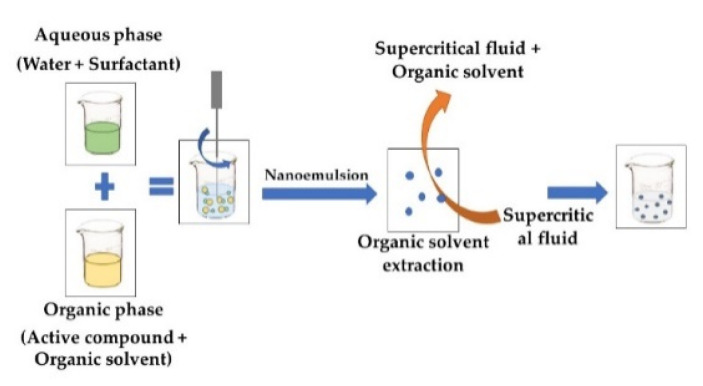
**MICROFLUIDIC** This method helps to reduce problems to obtain liposomes such as poly-disperse in size and lamellarity. It could be solved by applying different flow rate ratios (FRR) and the total flow rate (TFR) using a volume of fluids around 10^−9^–10^−8^ l within channels with dimensions of 10–100 mm. An aqueous buffer and solvent with lipid solution is necessary [51]	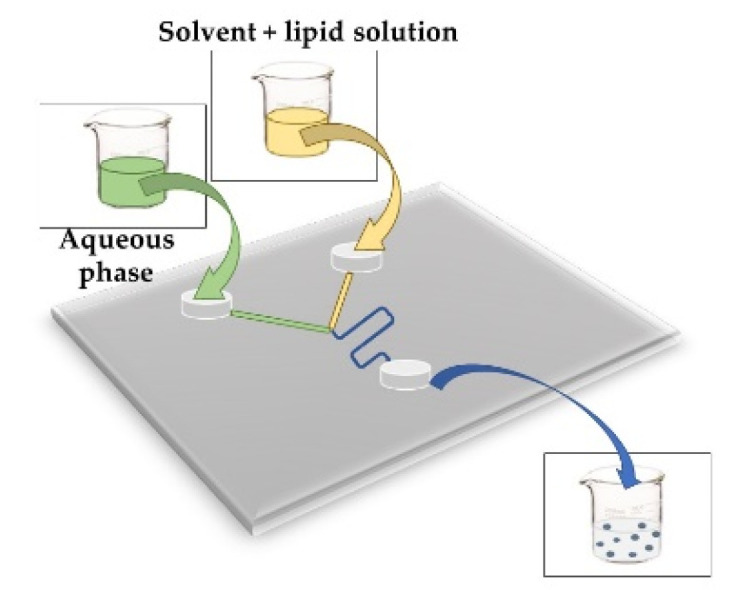

**Table 3:** Summary of nanosystems applied on meat and meat products and their antioxidant effect.

Nanosystem	Applications and Effects	Product	Ref.
Chitosan–vitamin C nanoparticle complexes CS–VC NP (23.2–82 nm)	Compared to controls and groups of meat treated only with CS or VC, the samples treated with CS–VC NP showed a good effect on delaying peroxidation by extending the induction period thanks to their antioxidant properties. CS–VC NP were incorporated onto the meat by dipping, then stored at 4 °C for 21 days.	Fresh ground meat	[57]
Chitosan–sodium tripolyphosphate nanoparticles CS–TPP NP	A CS–TPP NP solution was developed and applied to shrimp by vacuum tumbling. Quality characteristics were evaluated for 120 days during frozen storage (−20 °C). The CS-TPP NP treatments generated the highest reduction in lipid oxidation compared to other treatments under the same conditions since it showed the lowest TBARS value.	Shrimp	[58]
Cinnamon essential oil–chitosan nanoparticles CEO–CS NP (235.6 nm)	The efficacy of encapsulated CEO as a natural antioxidant that inhibits primary and secondary lipid oxidation in beef patties was assessed by measuring PV and TBARS values. The authors reported that CEO encapsulation can effectively retard lipid oxidation of beef patties even at low concentrations (0.05 and 0.1%) during storage at 4 °C for 8 days. At the end of storage, the 0.1 CEO–CS NP treatment had lower PV and TBARS values than the other treatments and the greatest resistance to oxidation.	Beef patties	[8]
Lyophilized pomegranate peel nanoparticles LPP NP (80 nm)	PV and TBARS values were lower in the LPP NP-treated samples than in the control for 15 days. LPP NP were added to the samples as part of the formulation of the meat-balls. The meat was stored at 4 °C for 15 days.	Meatballs	[59]
Chitosan–tripolyphosphate nanoparticles CS–TPP NP (12.6–321.5 nm)	CS-TPP NP added to the formulation of surimi exhibited an inhibitory role against lipid oxidation by reducing TBARS and the formation of conjugated dienes during 9 days of storage at 4 °C.	Surimi	[60]
Nerolidol nanospheres Ne NS (133 nm)	Nile tilapia fish were fed dietary supplements that contained 0.5 and 1 mL of nanoencapsulated nerolidol/Kg. Significant reductions in meat ROS and LPO levels were seen in the fish fed the 1.0-mL Ne NS/kg, suggesting a lower free radical content and reduced lipid damage, respectively.	Nile tilapia fish (*Oreochromis niloticus*)	[61]
Eugenol nanocapsules embedded with gelatin–chitosan (229.09 nm) Eug–Gel-CS NC	Eug-Gel-CS NC were applied to chilled pork meat by immersion. This treatment showed a lower rate of increase in TBARS than control samples. A fat-soluble free radical scavenger, Eug mediated hydroperoxide-protected muscles from oxidation and acted as an effective natural antioxidant by inhibiting lipid oxidation in the muscle. Storage temperature was 4 °C for 15 days.	Chilled pork meat	[62]
ε-polylysine nanoparticles with plant extracts ε-PLN	ɛ-PLN combined with a mixed extract (green tea, stinging nettle, olive leaf) can be used as a potential nitrite replacer in frankfurter-type sausages due to its effective antioxidant activity during refrigerated storage at 4 °C for 45 days. The authors reported that the sausages containing ε-PLN and the extract mixture had significantly lower TBARS values after 45 days. At the end of storage, samples of the sausage with ε-PLN had a TBARS value of 2.39, which is within acceptable limits (2–2.5 mg MDA/kg). Neither the meat itself nor the meat products tested showed rancidity.	Frankfurter-type sausages	[63]
Zinc nanoparticles Zn NP	Results indicated that feeding chicken broilers Zn NPs reduced the MDA content of breast meat, suggesting lower oxidative damage and lipid peroxidation due to improvement in the broilers’ antioxidant status.	Breast and thigh chicken fillets	[64]
Quercetin nanoparticles QT NPz	Five dietary treatments were formulated with increasing percentages of QT NPs (0, 100, 200, 300, and 400 mg/kg of diet). ROS production and malondialdehyde (MDA) content in muscle were reduced markedly at higher levels of dietary QT NP supplementation, suggesting a decrease in free radical contents and less lipid damage. Administering QT NP-enriched diets decreased ROS production by increasing cellular resistance to oxidative stress, causing a subsequent decrease in lipid peroxidation and higher numbers of healthy cells.	Nile tilapia fish (*Oreochromis niloticus*)	[65]

## Data Availability

Data is contained within the article.
